# Tumor lysis and acute renal failure in Burkitt's lymphoma: A review on pathophysiology and management

**DOI:** 10.4103/0971-4065.57102

**Published:** 2009-07

**Authors:** I. O. Senbanjo

**Affiliations:** Department of Paediatrics and Child Health, Lagos State University College of Medicine, Ikeja, Lagos State, Nigeria

**Keywords:** Acute renal failure, tumor lysis syndrome, Burkitt's lymphoma, hematologic malignancies, lymphoma, leukemia, management

## Abstract

Morbidity and mortality in patients with hematologic malignancies are significantly increased by development of acute renal failure. This is more likely in the developing world where facilities for renal replacement therapy are scarce. This review discusses the pathophysiology of acute renal failure due to tumor lysis syndrome in patients with Burkitt's lymphoma, the commonest hematological malignancy in the pediatric age group in sub-Saharan Africa, and evaluates the possible management options. Tumor lysis can also develop in association with other hematologic malignancies, both spontaneously and following treatment, and these principles are applicable in all such cases.

## Introduction

Although malnutrition and infectious diseases remain the major causes of childhood morbidity and mortality in the tropics and subtropics, the contributions of malignancies to childhood morbidity and mortality are rising apparently due to an increase in awareness and the improvement in immunization status.[1,2]

Burkitt's lymphoma (BL) is the most common childhood tumor found in the tropics and subtropics.[3] It was first described by Dr. Dennis Burkitt, a British surgeon, while he was working in Uganda.[4] Burkitt's lymphoma accounts for > 60% of childhood tumors in most parts of tropical Africa.[5] In Nigeria, it represents between 19.4–51.5% of all childhood tumors.[3,6] The tumors have the potential to affect almost every part of the body. Commonly affected sites are the abdomen, face, the central nervous system, and the thyroid gland.[3] In a study of the prevalence and etiology of acute renal failure in southwestern Nigeria, renal involvement by BL contributed 47.2% of the primary etiologies.[7]

BL is a prototype of high grade non-Hodgkin lymphoma characterized by high tumor burden. It is exquisitely chemosensitive, and responds well to treatment. Rapid cell lysis leads to release of massive amounts of breakdown products giving rise to the so called “tumor lysis syndrome” with acute renal failure (ARF). In developed countries, it is possible to achieve a 100% success rate in the management of BL complicated by ARF. However, in the tropics and subtropics, mortality is increased by factors such as a delay in presentation at the hospital, poverty, complicating septicemia, hypertension, and nonavailability of cytotoxic drugs and other cancer management facilities. Indeed, ARF and hypertension were shown to contribute significantly to morbidity and mortality in BL patients.[8] Therefore, an understanding of the pathophysiology and management of renal failure may contribute to a reduction in mortality. These principles are equally applicable in all high grade lymphomas and leukemias where the cell turn over rate is high.

## Pathophysiology

There are different mechanisms for the development of acute renal failure in BL patients. In addition to the acute tumor lysis syndrome (ATLS),[9] ARF in may arise from compressions of the renal tubules by the tumor,[10] infiltration of the kidney,[11] and toxic effects of anticancer drugs on the kidney.[9]

**Figure 1:**
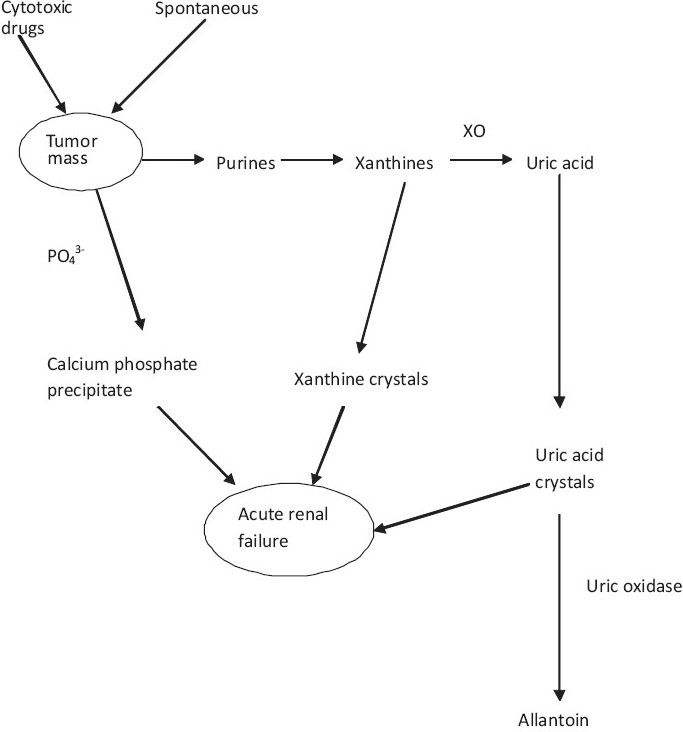
Pathophysiology of acute renal failure following tumor lysis syndrome. XO – Xanthine oxidase, PO_4_^3-^ - Phosphate

ATLS describes the metabolic derangements that may follow the initiation of cytotoxic therapy and rapid destruction of tumor cells. It may also occur spontaneously when the tumor outgrows its blood supply, leading to ischemia, necrosis, and release of cellular contents such as hypoxanthine, xanthine, uric acid, potassium, and phosphate into the extracellular space.[9,12] These metabolites can overwhelm the body's normal homeostatic mechanisms and form urinary crystals and precipitates that cause ARF. Three forms of these crystals are known which may occur in isolation or in combination [Figure 1].[9] They include: (i) calcium phosphate precipitates from hyperphosphatemia, (ii) uric acid crystals, and (iii) xanthine crystals. These crystals and precipitates block the renal tubules causing a reduction in the glomerular filtration rate (GFR) and hence, acute renal failure. There is also a direct toxic effect of uric acid on the renal parenchyma as well as a direct toxic effect of xanthine on the renal tubules, contributing to the development of ARF.[9]

Acute renal failure may also occur from intrinsic or extrinsic compression of the renal tubules. This may be due to direct infiltration and destruction of the renal tubules by malignant cells. There have been reports of acute renal failure occurring due to extrinsic compression of the bladder and ureters by large retrovesical Burkitt lymphoma.[11]

## Clinical Features

In addition to the features of the underlying disease, those complicated by ARF present with the following:

- Oliguria – This is defined as urinary output < 400 mL/m^2^/day.[7]
- Edema – This can present as facial edema which is usually worse in the morning but regresses towards evening. It can also present as ascites or bilateral, pitting, pedal edema.
- Abdominal swelling – This could be the result of a tumor mass in the abdomen, involvement of the liver, spleen, kidney, and ovaries, or due to ascites.
- Hypertension – This may arise from the compression of the renal artery by the tumor mass leading to the activation of the renin-angiotensin-aldosterone mechanism, causing sodium and water retention and hypertension. It could also be a manifestation of acute renal failure.[8]
- Proteinuria and Hematuria – In lymphomatous infiltration of the kidney, patients may present with proteinuria, hematuria, and ARF; this is usually an incidental finding.[9]
- There could be nonspecific symptoms such as nausea, vomiting, anorexia, diarrhea, lethargy, and seizure. This could be as a result of uremia or part of the features of ATLS.[13,14]
- Other features of ATLS – These are cardiac arrhythmias, muscle cramps, tetany, syncope, and possible sudden death. These are due to electrolyte imbalance, especially hypocalcemia and hyperkalemia.[13,14]

## Management

Tumor lysis syndrome and acute renal failure are important complications of any high grade lymphoma including BL. An anticipatory and aggressive approach is required for the prevention and management of these conditions.[12] Patients with advanced stages of the tumor as well as large tumor masses are at risk of renal failure.[15] It is therefore, important to carry out a comprehensive evaluation of renal functions especially those with advanced stage disease.

The conventional approach to the prevention and management includes:

### Allopurinol/Rasburicase

Allopurinol, a xanthine oxidase inhibitor, helps offset hyperuricemia arising from a high cell loss rate and therefore, prevent uric acid nephropathy.[9] The dosage is 10 mg/kg/day and is best started between 24 and 48 hours before the institution of chemotherapy. Its major shortcoming lies in the resultant accumulation of xanthine which also could form crystals in renal tubules, leading to renal failure.[9] The other limitations of allopurinol include hypersensitivity reactions, drug interactions, dosage adjustment in renal failure, and a response time of several days.[9] Therefore, a new approach to the prevention and treatment of uric acid nephropathy involves the use of the enzyme uricase, which catalyzes the oxidation of uric acid to the more soluble allantoin which is excreted by the kidneys.[9,15] Rasburicase is a polyethylene glycol-modified, recombinant uricase preparation that has been approved for use by the US-Food and Drug administration.[9] Unfortunately, this drug is not yet available for routine use in the developing world.

### Hydration

Patients with BL must be properly hydrated before and during chemotherapy. Therefore, at least 2–3 L/m^2^/day of fluid should be administered per day.[8,13] Adequacy of fluid administration should be judged by the urine output which should be in the range of 80–100 mL/m^2^/hour.[13] This promotes the renal excretion of uric acid and other metabolites and prevents toxic nephropathy. Adequate hydration with or without urinary alkalization have been shown to dramatically reduce the incidence of uric acid nephropathy.[9]

### Alkalinization of urine

In centers with adequate resources, the practice is to routinely hemodialyze (intermittent hemodialysis or continuous veno-venous hemodiafiltration) patients with ATLS and ARF.[16] Where this is lacking, the total fluid intake should be restricted to cover the insensible fluid loss and the volume of urine passed in the previous 24 hours given as 10% dextrose-in-water. The use of saline-containing fluid will lead to salt and water retention, and in turn, to intravascular congestion, circulatory overload, pulmonary edema, and raised blood pressure. Intravenous 8.4% sodium bicarbonate at the dosage of 0.5–1 mL/kg is added to this daily fluid requirement in those patients who have metabolic acidosis. Intravenous frusemide 2–4 mg/kg/day in 2–3 divided doses is also given in the first 24 hours preceding cytotoxic therapy. In addition to aiding the excretion of uric acid, sodium bicarbonate also helps in cellular ingress of potassium. It should be noted that alkalization of urine in the prevention and treatment of ATLS is currently not recommended.[14,17]

### Antihypertensives

Angiotensin-converting enzyme (ACE) inhibitors block the conversion of angiotensin I to a potent vasoconstrictor, angiotensin II, and can be used to treat renin-dependent hypertension.[8] They should, however, be used with caution in patients with ARF because of their potential to cause ARF in cases without preexisting renal disease.[18] Other antihypertensives that can be used include alphamethyldopa and calcium channel blockers. A calcium channel blocker which has a protective effect on the kidneys would be preferable in patients with ARF who need improved renal blood flow and glomerular filtration rate without any alteration of the filtration fraction.[19] In situations of severe high blood pressure, intravenous hydrallazine at the dosage of 0.2–0.5 mg/kg is given every six hours until the diastolic blood pressure falls to the upper limit of the range considered normal for the patient's age. The patient is further maintained in a normotensive state with either of the aforementioned antihypertensives.

### Cytotoxic therapy

The slow and cautious introduction of cytotoxic drugs helps reduce the metabolic load on the kidneys.[20] A large number of these drugs have toxic effects on the kidneys, thus causing ARF.[9] An example is methotrexate, which at high doses, may cause urinary obstruction secondary to intratubular precipitation.[9] In a comparative study of two treatment regimens, it was shown that the use of infusion of low-dose cyclophosphamide given on alternate days in addition to preemptive anti-TLS measures, was associated with better outcomes in patients with BL complicated by ARF compared to a high-dose multiple chemotherapy regimen.[20]

### Consensus Guidelines on the Management of ATLS

An expert panel from three consensus conferences held in Bologna, Italy between 20^th^ December, 2006 and 22^nd^ May, 2007 agreed that there is a need to categorize prevention and treatment of ATLS by separately grouping patients with high and low risk of developing ATLS.[13,14] The parameters for high and low risk are as defined by Cairo and Bishop.[17] Those with clinical or laboratory evidence of ATLS and those with a high risk of having ATLS are recommended to receive rasburicase and proper hydration. Those with a low risk of having ATLS are recommended to receive allopurinol and proper hydration. The basis for this difference is the ability of allopurinol to only cause a reduction in the formation, but not degradation, of uric acid, which implies a delay in the resumption of chemotherapy. Rasburicase, on the other hand, causes a rapid and complete degradation of uric acid to allantoin, potentially allowing a prompt continuation of chemotherapy.

## Conclusion

Acute renal failure complicating BL can be life-threatening. Efforts should therefore be made to prevent its occurrence through the use of allopurinol and proper hydration, with or without alkalization of urine. Where facilities for dialysis are not available for established cases of BL with acute renal failure, the use of allopurinol, treatment of hyperkalemia, and intermittent low-dose cytotoxic drugs result in better outcomes for these patients.
